# The Stockholm experience: interhospital transports on extracorporeal membrane oxygenation

**DOI:** 10.1186/s13054-015-0994-6

**Published:** 2015-07-09

**Authors:** L. Mikael Broman, Bernhard Holzgraefe, Kenneth Palmér, Björn Frenckner

**Affiliations:** ECMO Center Karolinska, Karolinska University Hospital, 171 76 Stockholm, Sweden; Department of Pediatric Surgery, Women’s and Children’s Health, Karolinska University Hospital, Solna, 171 76 Stockholm Sweden

## Abstract

**Introduction:**

In severe respiratory and/or circulatory failure, extracorporeal membrane oxygenation (ECMO) may be a lifesaving procedure. Specialized departments provide ECMO, and these patients often have to be transferred for treatment. Conventional transportation is hazardous, and deaths have been described. Only a few centers have performed more than 100 ECMO transports. To date, our mobile ECMO teams have performed more than 700 transports with patients on ECMO since 1996. We describe 4 consecutive years (2010–2013) of 322 national and international ECMO transports and report adverse events.

**Methods:**

Data were retrieved from our local databases. Neonatal, pediatric and adult patients were transported, predominantly with refractory severe respiratory failure.

**Results:**

The patients were cannulated in 282 of the transports, and ECMO was started in these patients at the referring hospital and then they were transported to our ECMO intensive care unit. In 40 cases, the patient was already on ECMO. Of the transports, 60 % were by aircraft, and the distances varied from 6.9 to 13,447 km. In about 27.3 % of the transports, adverse events occurred. Of these, the most common were either patient-related (22 %) or equipment-related (5.3 %). No deaths occurred during transport, and transferred patients exhibited the same mortality rate as in-hospital patients.

**Conclusions:**

Long- and short-distance interhospital transports on ECMO can be safely performed. A myriad of complications can occur, but the mortality risk is very low. The staff involved should be highly competent in intensive care, ECMO physiology and physics, cannulation, intensive care transport and air transport medicine. They should also be skilled in recognition of risk factors involved in these patients.

**Electronic supplementary material:**

The online version of this article (doi:10.1186/s13054-015-0994-6) contains supplementary material, which is available to authorized users.

## Introduction

For patients with refractory, severe respiratory or/and circulatory failure extracorporeal membrane oxygenation (ECMO) can be a lifesaving procedure. Only specialized departments can provide ECMO, and patients often have to be transferred for treatment. Conventional transportation is a high risk, and deaths have been described in the literature [1]. Published articles in this field are increasing, but the total number of transports worldwide remains unknown. The majority of authors describe diminishing numbers of transports over time [2–5]. However, the experiences in ECMO transportation seem to differ, as only two centers have reported a total of more than 100 ECMO transports: the University Medical Center at Regensburg, Germany [6], and the University of Michigan, Ann Arbor, MI, USA [7, 8]. In 2001, our unit published data collected from the first 5 years of operation of an ECMO transportation service [9].

ECMO treatment in Stockholm commenced in 1987 with neonatal patients. Today the ECMO Center Karolinska covers all age groups, with approximately 80–90 ECMO runs annually. As of November 2014, 936 patients have been treated, comprising 362 neonatal, 213 pediatric and 361 adult patients, ranging in weight from 1500 g to >160 kg and in age from 32 gestational weeks to 77 years. Since 2007, about 45 % of the case mix is adults, closely followed by the newborns, and pediatric patients constitute approximately 15 %. Our ECMO intensive care unit (ICU) is, to our knowledge, the only ICU worldwide that treats ECMO patients only. The ECMO transport service at Karolinska University Hospital was started in 1996.

The aim of this work is to give a comprehensive description of the national and international ECMO transports performed by our department between January 2010 and December 2013.

## Methods

We conducted a retrospective analysis of the ECMO department’s two internal databases. Because we conducted a retrospective quality control study, we did not require approval from the local ethics committee, nor were any of the patients approached concerning consent to participate, inasmuch as no data can be traced back to any specific individual. We present data for 4 consecutive years: 2010–2013. Fisher’s exact test was performed to compare mortality between primary ECMO transports and in-house ECMO patients. A *p* value less than 0.05 was considered significant.

### Extracorporeal membrane oxygenation criteria

Both pulmonary and cardiac ECMO were performed. Pulmonary ECMO was dominant (>90 % of the cases) because our experience is in pediatric intensive care. Patients are referred to us by telephone from another ICU or hospital to the ECMO physician on call, who decides if the patient fulfills the criteria for ECMO. The basic inclusion criteria were potentially reversible acute respiratory and/or cardiac failure. Acute respiratory failure equated to a ratio between partial pressure of oxygen in blood to fraction of inspired oxygen less than 80 mmHg (fraction of inspired oxygen [FiO_2_], 1.0) in adult and pediatric patients and an oxygenation index greater than 40 at FiO_2_ of 1.0 in neonates. Other criteria include Murray score above 3, peak inspiratory pressure greater than 35 cmH_2_O (pressure control), pressure amplitude in high-frequency oscillation ventilation greater than 55 cmH_2_O (high-frequency oscillation ventilation) and prolonged refractory hypercarbia with acidosis (pH <7.10). Regarding reversible acute cardiac failure acidemia and/or lactatemia, central venous oxygen saturation less than 55 %, cardiac index less than 2 L/min m^2^ or vasoactive score in a time perspective above 45–50 μg/kg min^−1^ favor ECMO.

The criteria per se are the same as for in-hospital patients, with the exception that if the physician at the referring hospital describes a patient whose condition is rapidly deteriorating, we mobilize earlier to avoid a need for cardiopulmonary resuscitation. The patient’s status is, of course, taken into consideration in every individual case. If the patient does not fulfill ECMO criteria, whether a potential ECMO candidate or not, continued telephone support will be given if requested. Each year, 200 to 250 patients who were not eligible for ECMO are supported this way each year. Support will be continued, and if the patient subsequently fulfills ECMO criteria, the mobile ECMO team is launched.

### Mobile extracorporeal membrane oxygenation team

For primary ECMO transports (where the patient is cannulated at the referring hospital by the mobile ECMO team), a team consisting of an ECMO physician (anesthetist and transport team leader), an ECMO specialist (ICU nurse) and a cannulating surgeon are at hand for emergency retrieval of patients requiring ECMO. Both the ECMO physician and ECMO specialist are required to have passed an accredited education plan developed by the Extracorporeal Life Support Organization (ELSO), Ann Arbor, MI, USA. A scrub nurse may participate in the team as decided by the surgeon, a resource otherwise provided by the referring hospital. The time from decision to go until departure is between 30 and 90 minutes, 24 h per day, 7 days per week, all year round. A prompt departure is of utmost importance for a timely arrival. Upon arrival to the referring hospital, a final assessment of the patient is performed by the ECMO physician in conjunction with the cannulating surgeon, who together decide if ECMO is appropriate and the mode of ECMO to be used.

Concerning secondary transports (where the patient is already on ECMO before retrieval), up to two ECMO physicians and up to two ECMO specialists perform the transport. If an emergency situation should occur, more than two professionals are preferred. Secondary transports involve another set of problems. The patient may be awake; cannulas often have been running for a while; there is a risk of coagulation disorder; and there may be degrees of organ failure. It is important to ensure that all of these issues are addressed, as safety is considered a high priority in ECMO transportation.

### Equipment

All transport equipment, including surgical instruments, is prepacked according to patient age in a storage facility at our clinic.

Until the autumn of 2011, Bio-Medicus 550 consoles (Medtronic, Tolochenaz, Switzerland) were used with a ROTAFLOW centrifugal pump (Maquet Cardiopulmonary, Hirrlingen, Germany) or Stöckert CAPS roller pumps (Stöckert, Munich, Germany). Thereafter the ECMO pump system used was the PediVas/CentriMag (Levitronix, Zurich, Switzerland), owing to increased safety compared with alternative systems. Transports were also carried out using the CARDIOHELP system (Maquet Cardiopulmonary).

All cannulations were peripheral. The following single-lumen cannulas were used for venovenous or venoarterial ECMO: Bio-Medicus 8–14 French (Fr), 15–21 Fr/18 cm, 23 Fr/25 cm and 17–29/Fr 50 cm (Medtronic); Fem-Flex II 8–14 Fr (Edwards Lifesciences Nordic AB, Malmö, Sweden); or Maquet Venous HLS 25 Fr/38 cm. If a dual-lumen catheter was needed for venovenous ECMO, an OriGen catheter of 12 Fr, 13 Fr (reinforced), 15 Fr or 18 Fr (OriGen Biomedical, Burladingen, Germany) was used in neonatal and pediatric patients, and an Avalon Elite 27- or 31-Fr catheter (Maquet) was used in adults. The oxygenators used were MEDOS HILITE 800 LT, 2400 LT or 7000 LT (Medos Medizintechnik, Stolberg, Germany) or QUADROX (Maquet). An Elisée 250 (ResMed, Moissy-Cramayel, France) or HAMILTON-T1 ventilator (Hamilton Medical, Bonaduz, Switzerland) was used during transport. Blood gases and activated clotting times were assessed by using an i-STAT system (Abbott Point of Care, Maidenhead, UK), and an IntelliVue X2 patient monitor (Philips Healthcare, Best, the Netherlands) was used for patient monitoring. The patient was placed on a LifePort stretcher (LifePort, Woodland, WA, USA) during transport, and a carrier has been developed incorporating the Levitronix console and pump motor, gas tubes, flowmeters, manometers, oxygenators and heaters (HICO-AQUATHERM 660; Hirtz & Co., Cologne, Germany). The carrier is easily locked into most ground ambulances and aircraft.

### Transport logistics

The ECMO physician handled all transport-related coordination between the functions involved and the referring hospital. Equipment and ECMO team were transferred by an emergency vehicle to the referring hospital or to an airport. The distance covered on ground was up to 300 km (185 miles) and was influenced by weather, local distances to and from airports and ambulance services at the referral hospital. After stabilization on ECMO, the patient was transported to ECMO Center Karolinska by the Stockholm County Ambulance Service’s mobile intensive care unit (MICU), which is an intensive care vehicle, or by a local ambulance to the closest airport, where an ambulance aircraft (Cessna Citation II; Graf Air, Bromma, Sweden) was waiting. The transport was then commenced to a Stockholm airport, from whence the MICU transferred the ECMO team and the patient to ECMO Center Karolinska. Occasionally, lack of beds meant that the patient had to be transported directly to an ECMO unit/thoracic ICU elsewhere by the mobile ECMO team.

## Results

The ECMO Center Karolinska is the principal tertiary referral center for Sweden and other European countries. To date, over 700 patients have been transported on ECMO by our transport organization. Approximately 59 % of transports were by fixed wing (of which 8 % were performed with an ambulance in a Hercules military aircraft; Lockheed Martin, Marietta, GA, USA), 5 % by helicopter transport and 36 % on the ground. In 2009, one death occurred during transport: a neonatal patient in septic shock on venovenous ECMO who developed cardiac failure during transport in the airplane. The majority of transports have been performed within Sweden, which is the fourth largest country in Europe, covering an area of 450,000 km^2^ (174,000 square miles) and having a population of 9.7 million. The distance from north to south is 1574 km (978 miles), and 15 % of the area is above the polar circle. Owing to geographic considerations, the development of an ECMO transportation service for long distances was mandatory.

Primary and secondary transports have been performed to and from other European countries, including Finland, Norway, Denmark, Iceland, Ireland, United Kingdom, France, Germany, Spain, Poland and the Czech Republic, as well as Egypt and Australia*.* A substantial portion of the newborns and children from Finland (40 patients) and Ireland (46 patients) in need of ECMO treatment were brought to our unit between 2001 and 2014. A total of 67 patients from other countries were primary transports to ECMO Center Karolinska, Stockholm for continued ECMO treatment.

Between 2010 and 2013, the mobile ECMO team was launched 387 times. The distances covered ranged from 6.9 to 13,447 km (4.3–8357 miles). There were 282 primary and 40 secondary interhospital ECMO transports performed. Transfers off ECMO were conducted in 21 cases under conventional ventilation to an ICU at the Karolinska University Hospital to be near the ECMO resource (see Additional file 1). In 44 cases, the patient had either clinically improved and therefore did not fulfill ECMO criteria or had died upon arrival of the ECMO team.

The majority of the transports (201 [62 %)] were conducted by air (200 by aircraft and 1 by helicopter), and 121 (38 %) were on the ground by ambulance. Almost 73 % of the primary ECMO transports brought the patient to ECMO Center Karolinska for continued ECMO treatment (see Additional file 2). All international transports were by fixed wing, and 80 % of these patients were taken to ECMO Center Karolinska, Stockholm.

The remainder of the transports consisted of primary transfers to other ECMO facilities because of either a lack of beds in our department or a request for us to perform a primary or secondary ECMO transport by another unit or hospital. Secondary ECMO transports also brought back our outpatients who had been allocated elsewhere initially owing to lack of beds (*n*=5).

The most common diagnosis in the pediatric and adult populations was severe refractory respiratory failure of infectious origin with or without septic shock (see Additional file 3). In the neonatal population, meconium aspiration syndrome, persistent pulmonary hypertension, sepsis and congenital diaphragmatic hernia were the most common reasons for ECMO transportation. No deaths occurred during our transports between 2010 and 2013, and no differences were seen in mortality rates in any age group or category (pulmonary, cardiac or extracorporeal cardiopulmonary resuscitation) compared with in-hospital patients. Regarding secondary transports, outcome data are not available, because these transports in part relate to patients retrieved and transported for treatment and/or intervention elsewhere and therefore were lost to follow-up.

Complications during transport were possible to extract from 300 of the 322 primary and secondary transports performed on ECMO. Missing data were due to either lack of transport documentation in the patient chart or the fact that the patient record not could be located. Incidents were reported in 82 cases (27.3 %). In 14 cases, more than one event occurred, with a total number of 94 adverse events. These could be categorized into five major groups: patient, staff, equipment, vehicle and environment. Most adverse events occurred in the patient category (22 %), where loss of tidal volume (12.7 %) was the most common (see Additional file 4).

## Discussion

Most published data support the feasibility of interhospital transports on ECMO [2–5, 10–12]. The mortality rate during ECMO transport is low, reportedly 0.5 % [8]. In our presented cases, no deaths occurred, and only one death has happened since the start of our transport organization, now comprising more than 700 transports on ECMO. During the observed period, we performed a total of 322 ECMO transports: 282 primary and 40 secondary. Seventy-six patients (27 %) in the primary transport cases were not transported to our ECMO ICU, owing to lack of beds in our unit. Regarding secondary transports, 80 % of these were taken to our ICU. The remaining 20 % were either transport missions between thoracic ICUs in northern Europe or patients in our institution who were allocated to an ECMO-performing ICU abroad to enable space for acute admittance of critically ill neonatal or pediatric patients too unstable to survive a prolonged interhospital transport.

Because transports on ECMO are highly complex, the staff involved should be experienced in prehospital emergency medicine, intensive care, ECMO physiology, ECMO technology and ECMO cannulation [13, 14]. Different health care systems have their own strategies for how to organize and staff an ECMO retrieval organization [6, 15–18, 11, 12]. Despite the challenges posed by our geographic location (long distances, freezing temperatures to −30 °C, reduced sunlight hours) our system is efficient and safe. Our teams are highly experienced and able to deal with complications. Most transports carry minor problems within themselves, predominantly of negligible risk to the patient’s safety. However, they do require resolution, and logistical problems will engage the transport team leader. Unfortunately, in some cases, these are due to ambulance services’ not being aware of what ECMO is and even less what equipment and risks are inherent in an ECMO transport.

Complications during transport are predominantly patient-related. In our practice, the ventilator pressures and peak inspiratory and positive end-expiratory pressures are reduced to let “the lung rest.” The reason for this is not that a loss of tidal volume is required, but rather to keep the lung open during transport as a way of rescue if the ECMO system fails. However, a portion of the cases where the tidal volume is lost is not only a cause of the illness and its dynamics but also our approach to the ventilator settings.

There are very few published studies on complications during ECMO transport [8, 12]. Adverse events should be expected. The data we present should be interpreted as an underestimation of what really happens “out there.” The forthcoming ELSO guidelines concerning ECMO transport should provide definitions and a form to be submitted to the ELSO Registry for every ECMO transport. An international system of reporting to ELSO should be uniform and produce comparable and reliable data.

### Safety and quality issues

There are no published data to support the minimum numbers a mobile ECMO team should perform. Inferences could be drawn from parallel recent reports on ECMO treatments per center and per year. Since the influenza A(H1N1) pandemic, the number of ECMO centers has increased substantially. However, the meaning of “ECMO Center” has not been defined. With regard to adults, a consensus statement from the International ECMO Network proposes 20 cases incorporating at least 12 respiratory adult ECMO cases to be required on annual basis [19]. For neonatal and pediatric patients, Freeman et al. [20] have shown a minimum annual caseload of at least 20 and 22, respectively, to increase survival rate compared with low-volume centers. Twenty annual cases is considered the low number for an adequate learning curve and to maintain ECMO competence. In a recent publication, Barbaro and co-workers showed that ECMO centers with more than 30 annual adult ECMO treatments had a significantly lower ECMO mortality than units with fewer than 6 cases per year. There was also a significant variability in mortality rates among units that could not be explained by the number of cases treated. This volume–mortality association might favor a policy to continue and expand treatment at the experienced centers or even to centralize ECMO treatment instead of starting an ECMO program at another hospital [21]. Others have reported support of transfers to a regional ECMO center [16, 18, 19, 22]. The United Kingdom, Australia and Italy organize their ECMO services in this manner, with regional centers with a retrieval service.

Only a few study reports have included morbidity figures, but, as seen in our unit, the population exposed to an ECMO transport has about the same mortality rate as the corresponding non-transferred patients at a given ECMO center [7, 11, 18].

The future rationale would be that one well-trained transport organization performs ECMO transport at a high-volume ECMO unit as part of their service for a particular region [18, 19, 22]. This does not overrule the possibility of lower-volume hospitals commencing a patient on ECMO in an emergency and subsequently having the patient transported to a regional ECMO center [19]. Some Finnish university hospitals less experienced in ECMO proceed in this manner. In our 4-year data, 13 patients died before ECMO was commenced. A few of these lives might have been saved in such a system. It is also important to remember that there is an educational aspect to this; we have to teach our colleagues in referring hospitals about early detection and referral of the potential ECMO candidate.

## Conclusions

Long- and short-distance interhospital transports on ECMO can be performed safely. The staff involved should be highly competent in intensive care, ECMO physiology and physics, ECMO cannulation, intensive care transport and air transport medicine (if applicable). Importantly, they should be aware of risk factors involved in transporting these patients and management of complications.

Because ours is the largest institute conducting national and international ECMO transport, we argue that in times of growing ECMO demand, units should perform more than 20–30 ECMO runs per year. Such organizations will bring a significantly higher survival rate and total costs and resources spent will be better used for the population they serve. It should be emphasized that the patient should, whenever possible, be transferred for treatment at a high-volume ECMO center; hence, an efficient ECMO transport service is needed. The numbers of ECMO centers that provide predominantly respiratory support should be kept to 1 per 5–10 million population if offering the ECMO service to all three age groups, or to 1 per 8–15 million if supporting only adults. An ECMO transport service should be integrated with that specific ECMO center offering service on demand on a 24/7 basis*.*

## Key messages

- Interhospital transports on ECMO of the critically ill patient is safe.
- Safety increases if interhospital transport on ECMO is performed by a high-volume, highly experienced mobile ECMO transport organization.
- Complications during these hazardous transports are to be expected.

## Additional files

Additional file 1:
**ECMO team launches between 2010 and 2013.** Additional file 1 shows the type of transport and place for commencement of ECMO treatment for interhospital ECMO transport between 2010 and 2013. *Primary ECMO transport*: The patient is cannulated and ECMO started at referring hospital before transport. *Secondary ECMO transport*: The patient already is cannulated when the ECMO team arrives. Additional file 2:
**Origin of ECMO patient and primary unit for commenced treatment.** Absolute numbers are shown with frequencies in percent (%). Additional file 3:
**Primary diagnosis for ECMO retrieval.** Additional file 3 shows the numbers and frequencies of primary cause for ECMO retrieval within each age group and category. Diagnosis followed by number in *italic* expresses subdiagnosis within any of the primary diagnoses. In 199 of the 202 primary ECMO transports where treatment was commenced at our department, the primary diagnosis could be retrieved from our database. *ECPR* extracorporeal cardiopulmonary resuscitation; *P/F ratio*, ratio between partial pressure of oxygen in blood to fraction of inspired oxygen, calculated as PaO_2_ (mmHg)/FiO_2_ (%/100); *OI* Oxygenation index, calculated as [(FiO_2_ × Mean Airway Pressure (cmH_2_O)]/PaO_2_ (mmHg); *BctPneu* bacterial pneumonia; *VirPneu* viral pneumonia; *ARF* acute respiratory failure; *ARDS* acute respiratory distress syndrome; *Bridge*: bridge to lung transplant; *PCPneu Pneumocystis jirovecii* (*Pneumocystis carinii*) pneumonia; *MAS* meconium aspiration syndrome; *CDH* congenital diaphragmatic hernia; *PPHN* persistent pulmonary hypertension in the newborn; *PFC* persistent fetal circulation; *CAD* capillary alveolar dysplasia; *AMI* acute myocardial ischemia. Additional file 4:
**Incidents during ECMO transports.** Additional file 4 shows the numbers and frequencies of chart notes concerning incidents and adverse events during ECMO transports between 2010 and 2013. Of 322 transports on ECMO, journals were recovered in 300 cases (93.2 %).

## Abbreviations

ECMO: Extracorporeal membrane oxygenation
ELSO: Extracorporeal Life Support Organization
FiO_2_: Fraction of inspired oxygen
ICU: Intensive care unit
MICU: Mobile intensive care unit (i.e., ambulance truck)
OI: Oxygenation index, calculated as [FiO_2_ (%) × 100 × mean airway pressure (cmH_2_O)]/pO_2_ (mmHg)
