# Supplementary figures and images for: The Stockholm experience: interhospital transports on extracorporeal membrane oxygenation
Source: Crit Care. 2015 Jul 9;19(1):278. doi: 10.1186/s13054-015-0994-6 (PMC4498561; doi:10.1186/s13054-015-0994-6)

**
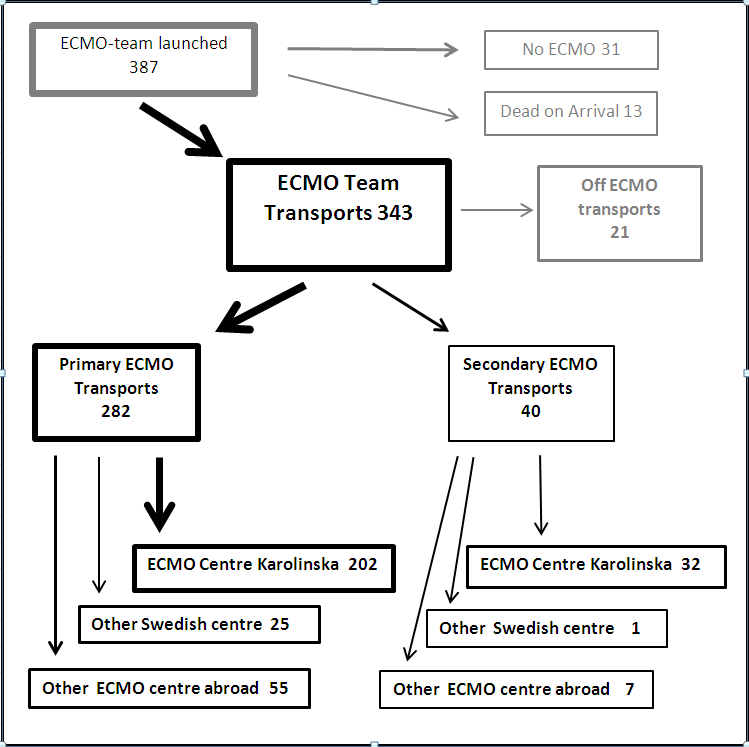
**

Supplement: Additional file 1: — ECMO team launches between 2010 and 2013. Additional file 1 shows the type of transport and place for commencement of ECMO treatment for interhospital ECMO transport between 2010 and 2013. Primary ECMO transport: The patient is cannulated and ECMO started at referring hospital before transport. Secondary ECMO transport: The patient already is cannulated when the ECMO team arrives. [file 13054_2015_994_MOESM1_ESM.doc]
