# Supplementary material for: The Stockholm experience: interhospital transports on extracorporeal membrane oxygenation
Source: Crit Care. 2015 Jul 9;19(1):278. doi: 10.1186/s13054-015-0994-6 (PMC4498561; doi:10.1186/s13054-015-0994-6)
Supplement: Additional file 2: — Origin of ECMO patient and primary unit for commenced treatment. Absolute numbers are shown with frequencies in percent (%). [file 13054_2015_994_MOESM2_ESM.doc]

| **To**  **From** | **ECMO Center Karolinska** | **Other ECMO-unit, Sweden** | **ECMO-unit abroad** |
| --- | --- | --- | --- |
| **Stockholm county n=62** | **45(73%)** | **9(14)** | **8(13)** |
| **Rest of Sweden n=169** | **130(77)** | **15(9)** | **24(14)** |
| **Other Country n=51** | **27(53)** | **1(2)** | **23(45)** |
| **All n=282** | **202(72)** | **25(9)** | **55(19)** |
