# Supplementary material for: The Stockholm experience: interhospital transports on extracorporeal membrane oxygenation
Source: Crit Care. 2015 Jul 9;19(1):278. doi: 10.1186/s13054-015-0994-6 (PMC4498561; doi:10.1186/s13054-015-0994-6)
Supplement: Additional file 3: — Primary diagnosis for ECMO retrieval. Additional file 3 shows the numbers and frequencies of primary cause for ECMO retrieval within each age group and category. Diagnosis followed by number in italic expresses subdiagnosis within any of the primary diagnoses. In 199 of the 202 primary ECMO transports where treatment was commenced at our department, the primary diagnosis could be retrieved from our database. ECPR extracorporeal cardiopulmonary resuscitation; P/F ratio, ratio between partial pressure of oxygen in blood to fraction of inspired oxygen, calculated as PaO2 (mmHg)/FiO2 (%/100); OI Oxygenation index, calculated as [(FiO2 × Mean Airway Pressure (cmH2O)]/PaO2 (mmHg); BctPneu bacterial pneumonia; VirPneu viral pneumonia; ARF acute respiratory failure; ARDS acute respiratory distress syndrome; Bridge: bridge to lung transplant; PCPneu Pneumocystis jirovecii (Pneumocystis carinii) pneumonia; MAS meconium aspiration syndrome; CDH congenital diaphragmatic hernia; PPHN persistent pulmonary hypertension in the newborn; PFC persistent fetal circulation; CAD capillary alveolar dysplasia; AMI acute myocardial ischemia. [file 13054_2015_994_MOESM3_ESM.doc]

| **RESPIRATORY** |  |  | **CARDIAC** |  |  |
| --- | --- | --- | --- | --- | --- |
| N = 178 | **n** | **%** | N = 13 | **n** | **%** |
| **Adult** | 93 | 91,2 | **Adult** | 5 | 4,9 |
| Age (mean±1SD) | 48,6±16,5 |  | Age (mean±1SD) | 57,2±18,7 |  |
| median (min/max) | 51,2 (18,3-77,0) |  | median (min/max) | 62,2 (25,5-71,1) |  |
| Male gender | 59 | 63,4 | Male gender | 4 | 80,0 |
| ***Diagnoses*** |  |  | ***Diagnoses*** |  |  |
| Sepsis | 35 | 34,3 | AMI | 3 | 2,9 |
| BctPneu | 32 | 31,4 | Pulm emboli | 1 | 1,0 |
| *H1N1* | *11* | 10,8 | Survival to discharge | 4 | 80,0 |
| VirPneu | 9 | 8,8 |  |  |  |
| Aspiration | 6 | 5,9 |  |  |  |
| ARDS | 8 | 7,8 | **Pediatric** | 3 | 9,7 |
| ARF | 2 | 2,0 | Age (mean±1SD) | 4,9±8,1 |  |
| *Trauma* | *5* | 4,9 | median (min/max) | 0,2 (0,2-14,3) |  |
| Bridge | 1 | 1,0 | Male gender | 1 | 33,3 |
| P/F (n=73) | 59,3±25,6 |  | ***Diagnoses*** |  |  |
| median (min/max) | 55,5 (30-240) |  | Adenovirus | 1 | 33,3 |
| Survival to discharge | 65 | 70,0 | Survival to discharge | 3 | 100,0 |
|  |  |  |  |  |  |
| **Pediatric** | 25 | 80,6 | **Neonatal**  N | 5 | 7,7 |
| Age (mean±1SD) | 5±5,17 |  | Male gender | 2 | 40,0 |
| median(min/max) | 3 (0,2-17,4) |  | ***Diagnoses*** |  |  |
| Male gender | 14 | 56,0 | Adenovirus | 2 | 40 |
| ***Diagnoses*** |  |  | Valve/heart | 2 | 40 |
| VirPneu | 11 | 35,5 | Other | 1 | 20 |
| ARDS | 4 | 12,9 | OI | (26 and 31) |  |
| Sepsis | 3 | 9,7 | Survival to discharge | 4 of 5 | 80 |
| BctPneu | 3 | 9,7 |  |  |  |
| *H1N1* | *3* | 9,7 | **ECPR** |  |  |
| PCPneu | 2 |  | N = 8 | **n** | **%** |
| Aspiration | 2 | 6,5 | **Adult** | 4 | 3,9 |
| *Trauma* | *1* | 4,0 | Age (mean±1SD) | 54,2±6,2 |  |
| *Leukemia* | *1* | 4,0 | median (min/max) | 55,5 (45,7-60,4) |  |
| P/F | 58,5±20,6 |  | Male gender | 3 | 75,0 |
| median (min/max) | 58,0 (22,5-105) |  | Survival ECMO | 0 | 0 |
| Survival to discharge | 23 | 92,0 | Survival to discharge | 0 | 0 |
|  |  |  |  |  |  |
| **Neonatal** | 60 | 92,3 | **Pediatric** | 4 | 12,9 |
| Male gender | 29 | 48,3 | Age (mean±1SD) | 4,82±5,10 |  |
| ***Diagnoses*** |  |  | median | 5 |  |
| MAS | 28 | 43,1 | Male gender | 2 | 50,0 |
| PPHN/PFC | 10 | 15,4 | drowning | 1 | 25,0 |
| Sepsis | 8 | 12,3 | Survival to discharge | 1 | 25 |
| CDH | 7 | 10,8 |  |  |  |
| VirPneu | 3 | 4,6 |  |  |  |
| Aspiration | 1 | 1,5 |  |  |  |
| CAD | 2 | 3,1 |  |  |  |
| Other | 1 | 1,5 |  |  |  |
| OI±1 SD (min-max) | 66,9±35,1 (26-184) |  |  |  |  |
| Survival to discharge | 48 | 80,0 |  |  |  |
