# Supplementary material for: The Stockholm experience: interhospital transports on extracorporeal membrane oxygenation
Source: Crit Care. 2015 Jul 9;19(1):278. doi: 10.1186/s13054-015-0994-6 (PMC4498561; doi:10.1186/s13054-015-0994-6)
Supplement: Additional file 4: — Incidents during ECMO transports. Additional file 4 shows the numbers and frequencies of chart notes concerning incidents and adverse events during ECMO transports between 2010 and 2013. Of 322 transports on ECMO, journals were recovered in 300 cases (93.2 %). [file 13054_2015_994_MOESM4_ESM.doc]

|  | **n** | **%** |
| --- | --- | --- |
| **Transports with incidents** | **82** | **27.3** |
| **Transports witn > 1 incident** | **14** | **4.7** |
| **Total no of incidents** | **94** |  |
| **Patient related** | **66** | **22.0** |
| **Loss of tidal vol** | 38 | 12.7 |
| *flooding of lung* | *3* | *1.0* |
| **Bleedings** | 8 | 2.7 |
| *cannulation site* | *4* | *1.3* |
| *lung* | *3* | *1.0* |
| *nose* | *1* | *0.3* |
| **Hypoglycemia** | 1 | 0.3 |
| **Hyperkalemia** | 1 | 0.3 |
| **Bradycardia** | 1 | 0.3 |
| **Hypothermia** | 1 | 0.3 |
| **Thrombocytosis** | 1 | 0.3 |
| **Arousal** | 1 | 0.3 |
| **Cardiac stun** | 5 | 1.7 |
| **Leg ischemia** | 1 | 0.3 |
| **Vagal reflex/secretions** | 1 | 0.3 |
| **Hypovolemia** | 3 | 1.0 |
| **Circulatory instability** | 1 | 0.3 |
| **Pericardiac cannulation** | 1 | 0.3 |
| **Recirculation (VV ECMO)** | 1 | 0.3 |
| **Loss of arterial line** | 1 | 0.3 |
| **Equipment/technical** | **16** | **5.3** |
| **Clotting of ECMO-system** | 2 | 0.7 |
| **Canulla clot** | 1 | 0.3 |
| **Oxygenator clot** | 1 | 0.3 |
| **Broken lab device** | 4 | 1.3 |
| **Syringe pump failure** | 2 | 0.7 |
| **Broken heater/hose** | 3 | 1.0 |
| **Broken oxygen hose** | 1 | 0.3 |
| **Broken ventilator hose** | 1 | 0.3 |
| **Loss of power supply to pump** | 1 | 0.3 |
| **Staff flaw** | **2** | **0.7** |
| **ECMO-system forgotten** | 1 | 0.3 |
| **Pump head forgotten** | 1 | 0.3 |
| **Vehicle/transportation** | **7** | **2.3** |
| **Wrong ambulance** | 2 | 0.7 |
| **Ambulance traffic accident** | 2 | 0.7 |
| *colliding w wildlife* | *2* | *0.7* |
| **Ambulance; no electricity** | 1 | 0.3 |
| **Change in destination** | 1 | 0.3 |
| **No transport aft. delivery** | 1 | 0.3 |
| **Environment** | **3** | 1.0 |
| **Reload in low amb. temp.** | 1 | 0.3 |
| **Freezing of iv lines** | 2 | 0.7 |
